# Integrating anatomical priors and clinical semantics for MRI-based diagnosis and care support in Alzheimer's disease

**DOI:** 10.3389/fnins.2026.1852056

**Published:** 2026-07-01

**Authors:** Xin Huang, Meining Zhang, Fang Chen

**Affiliations:** 1 Department of Neurology, Dazhou Central Hospital, Dazhou, China; 2 Department of Nursing, Dazhou Central Hospital, Dazhou, China

**Keywords:** Alzheimer's disease, anatomical priors, cross-modal alignment, diagnostic text, multimodal learning, structural MRI

## Abstract

Alzheimer's disease (AD) is a progressive neurodegenerative disorder, and magnetic resonance imaging (MRI) has become an important tool for its auxiliary diagnosis because it can reveal key structural abnormalities, including hippocampal and parahippocampal atrophy, ventricular enlargement, temporal cortical degeneration, and gray matter loss. However, reliable stage-aware classification remains challenging because current methods are still limited in handling weak anatomical boundaries, low-contrast lesions, subtle inter-stage differences, and the semantic gap between neuroimaging features and clinical descriptions. To address these issues, we propose AFCG-Net, a diagnosis-guided and frequency-aware cross-modal network for joint MRI-text diagnosis. The framework consists of three stages: anatomy-guided visual encoding, cross-modal semantic alignment, and gated fusion with anatomical priors. Specifically, ASFG enhances visual representations through multi-scale modeling and low frequency-guided refinement, CASF strengthens the consistency between clinical semantics and imaging features, and AGDF performs anatomy-guided deep fusion to improve both discriminability and interpretability. Experiments on a combined cohort of 4,197 original MRI-text samples, including 2,106 self-collected samples and 2,091 ADNI samples, show that AFCG-Net achieves Precision, Recall, and F-score values of 96.3%, 96.5%, and 95.8%, respectively. The proposed method achieves the best results in the Mild dementia and Moderate dementia categories, while also showing stronger performance in the more challenging Non-dementia and Very Mild dementia categories. These results suggest that AFCG-Net provides an effective and interpretable multimodal solution for AD-assisted diagnosis.

## 1 Introduction

Alzheimer's disease (AD) is a progressive neurodegenerative disorder that can cause early decline in memory, reasoning, and behavioral function, and, as the disease advances, imposes a substantial burden on both individuals and healthcare systems (Halliday, 2017; Sakato et al., 2024). Structural magnetic resonance imaging (MRI) (Grothe et al., 2016), owing to its non-invasive nature, high spatial resolution, and sensitivity to anatomical brain changes, has become an important imaging modality for AD-assisted diagnosis (Berron et al., 2022). It is capable of characterizing key alterations, including hippocampal and parahippocampal atrophy, ventricular enlargement, temporal cortical thinning, and gray matter volume loss (Xu et al., 2024). At the same time, imaging abnormalities in AD do not usually appear as explicit lesions with clear boundaries (Morais-Ribeiro et al., 2024); rather, they are more often manifested as weak-boundary, low-contrast, cross-scale, and continuously evolving anatomical degeneration, particularly in the early stage, where substantial overlap with normal aging is commonly observed (Scheltens, 1999; Hechkel and Helali, 2025). In real-world clinical settings, these challenges are further amplified by variations in scanning devices, acquisition protocols, image quality, class imbalance, and limited inter-rater consistency in manual interpretation. Moreover, the clinical case text, which contains information such as disease history, cognitive scale scores, and physicians' diagnostic impressions, is inherently high-level in semantics and lacks explicit spatial correspondence (Ai et al., 2025). Against this background, the central problem addressed in this study is how to jointly model MRI and clinical text under the coexistence of low contrast, weak boundaries, class imbalance, and semantic heterogeneity, so as to establish an AD staging framework that can stably characterize multi-scale anatomical degeneration while maintaining good generalizability (Dounavi et al., 2022).

Motivated by these challenges, existing studies have gradually shifted from traditional machine learning toward automated MRI (Viola et al., 2015)analysis centered on deep learning, and have made sustained progress in areas such as 2D/3D convolutional modeling, transfer learning, lightweight architectures, and multimodal fusion (Tufail et al., 2020; Irfan et al., 2023). Overall, these methods have demonstrated that deep MRI-based representations can effectively support automated AD staging, particularly showing strong potential in the recognition of middle and later disease stages (Yang et al., 2026). Nevertheless, the performance gains achieved by current studies still rely largely on single visual branches, extensive preprocessing, and specific data distributions, while explicit modeling of the continuous anatomical degeneration and cross-scale structural variations in Alzheimer's disease remains insufficient (Palani et al., 2026). At the same time, the generalizability of existing models across scanners, acquisition protocols, and populations is still limited, and confusion between adjacent early stages—especially between normal and very mild cases—remains the most prominent challenge (Dharshini et al., 2026). More importantly, current high-performing results are often accompanied by strong data dependence and weak mechanistic interpretability (Zhang et al., 2025), which to some extent restricts the clinical credibility and practical utility of these models. In other words, the key challenge is no longer whether automated classification can be achieved, but rather how to realize stable, interpretable, and fine-grained discrimination for early-stage AD under complex clinical conditions (He et al., 2025).

Based on the above limitations, further improving AD-MRI staging performance does not primarily depend on simply stacking deeper classifiers, but rather on simultaneously addressing three tightly coupled questions. First, how can local, regional, and global anatomical degeneration patterns be stably extracted from weakly informative MRI representations (Burns et al., 2026) Second, how can clinical text provide effective disease-semantic constraints for image understanding, rather than introducing spurious alignment (Gillis et al., 2025) Third, how can deep fusion be achieved between anatomical priors and cross-modal semantics under controllable noise conditions (Hassanzadeh et al., 2024) This judgment is grounded in the fact that imaging abnormalities in AD are essentially characterized by continuous degeneration across brain regions such as the hippocampus, ventricles, and cortex, rather than isolated abnormalities (Uğuz, 2025). At the same time, existing studies have repeatedly shown that early stage misclassification is primarily due to the substantial overlap between normal aging and subtle pathological alterations (Ferrer, 2022). These observations suggest three methodological requirements: a visual encoder that combines multi-scale spatial modeling with frequency-domain purification, a cross-modal alignment mechanism guided by global disease semantics rather than object–location matching, and an anatomy-constrained gated fusion module for collaborative multi-source modeling without amplifying spurious textures and noise.

Based on the above analysis, we propose AFCG-Net (Anatomy-guided Frequency-aware Cross-modal Gated Network) for joint MRI–text diagnosis of Alzheimer's disease (Yasin et al., 2025; Sandaruwan et al., 2025). Specifically, ASFG enhances discriminative MRI representations through multi-scale atrophy modeling and low-frequency-guided purification, CASF performs frequency-aware coarse semantic alignment under the guidance of global clinical semantics, and AGDF achieves stable gated fusion of anatomical priors, semantic information, and local details under anatomical constraints. Together, these three modules form a unified framework for anatomy-guided visual encoding, cross-modal semantic constraint, and structurally constrained deep fusion, with the final staging result produced by a classification head. Owing to its clear modular design and plug-and-play capability, AFCG-Net can be readily integrated into existing MRI classification or multimodal diagnostic systems, demonstrating practical and clinical potential.

The main contributions of this study are summarized as follows:

1. We propose AFCG-Net for Alzheimer's disease MRI staging, and construct a unified multimodal diagnostic framework that integrates anatomical structural priors, clinical text semantics, and a gated deep fusion mechanism.
2. We develop the ASFG visual encoding module, which enhances discriminative feature extraction from weak-boundary and low-contrast MRI through multi-scale atrophy modeling and low-frequency-guided purification.
3. We design the CASF cross-modal alignment module, which stably injects clinical text semantics through frequency-aware coarse semantic alignment and strengthens the consistency between multi-scale anatomical patterns and disease semantics.
4. We propose the AGDF deep fusion module, which constrains cross-modal feature fusion with anatomical structural priors, enabling collaborative modeling of structural information, disease semantics, and local details, and thereby improving the robustness and interpretability of the model.

## 2 Related work

### 2.1 Automatic classification of Alzheimer's disease based on MRI

Automatic diagnosis of Alzheimer's disease (AD) based on magnetic resonance imaging (MRI) has evolved from conventional machine learning paradigms toward deep representation learning. Early studies primarily employed 2D or 3D convolutional neural networks (CNNs) to extract local or regional neurodegenerative patterns from structural MRI, such as hippocampal atrophy, ventricular enlargement, cortical thinning, and gray-matter loss, achieving promising performance in AD classification and staging tasks. With the advancement of model architectures, Transformer-based models, hybrid CNN–Transformer frameworks, and lightweight networks have been increasingly introduced to enhance long-range dependency modeling and computational efficiency. For example, Xin et al. (2023) proposed ECSNet, which integrates CNNs with Swin Transformer and adopts a 2.5D subject-level strategy together with a dual-stream architecture to encode 3D sMRI information into 2D feature representations, thereby reducing computational complexity while improving the representational capacity of structural MRI. Zhang et al. (2024a) further developed a hierarchical attentive functional brain network within a Transformer framework, where sparse attention and node-merging mechanisms are employed to adaptively model multi-scale brain connectivity structures, providing new insights into early AD diagnosis and brain-network biomarker identification. In addition, MRI classification methods based on transfer learning, attention enhancement, and graph-structured modeling have demonstrated the ability of deep models to effectively capture AD-related anatomical alterations. Nevertheless, most existing approaches remain primarily dependent on a single imaging modality, making their performance susceptible to variations in scanning protocols, dataset distribution shifts, and the subtle or ambiguous disease boundaries at early stages. More importantly, these methods are often unable to fully exploit the complementary diagnostic cues embedded in clinical records, genetic profiles, or cognitive assessments.

### 2.2 Multimodal feature representation and fusion methods

To overcome the limitations of single imaging modalities, multimodal AD diagnosis has attracted increasing research attention in recent years. These methods typically integrate MRI with APOE genotype, demographic variables, or other clinical information to improve the model's ability to characterize disease heterogeneity and subtle pathological changes at early stages. Qiu et al. (2024) proposed MDL-Net, which jointly utilizes MRI and PET data and enhances the representation of AD-related brain regions through disease-induced region-aware learning and latent subtree learning. Nan et al. (2022) established a reproducible multi-class evaluation framework to systematically investigate the effects of different modality combinations and task settings on AD diagnostic performance. Zhang et al. (2024b) developed a feature-aware multimodal framework that integrates low-dimensional SHAP-based feature selection, cross-modal attention, graph convolutional networks, and an automatic fusion strategy, thereby preserving multimodal correlations while adaptively optimizing fusion weights. More recently, Liu et al. (2026) combined sMRI with APOE genotype and improved the discrimination among CN, MCI, and AD through spatial enhancement, multi-scale feature fusion, and an abstract patch module. NeuroNet-AD proposed by Rahman et al. (2025) further reflects the growing potential of multimodal deep learning for multi-class AD diagnosis. Beyond direct multimodal fusion, recent studies have also explored missing-modality synthesis or multimodal guidance to reduce dependence on PET. For example, Sun et al. (2024) inferred PET-related disease information from single-modal MRI, while Mallya and Hamarneh (2022) used a diagnostically stronger but less feasible modality to guide models that require only accessible inputs at inference. These studies provide practical solutions for screening scenarios in which PET is unavailable. Nevertheless, many existing multimodal methods still rely on feature concatenation, handcrafted fusion rules, or task-specific modality combinations, and the non-linear relationship modeling and semantic alignment among heterogeneous modalities remain insufficiently explored. To address this gap from a complementary perspective, our study integrates structural MRI, routinely available clinical text, and anatomical priors, without requiring PET acquisition or PET synthesis during inference.

### 2.3 Multimodal interaction and clinical semantic modeling

To further model the interactions among heterogeneous modalities, attention mechanisms and cross-modal learning have been widely explored in AD diagnosis. Tang et al. (2023) proposed CsAGP, which employs a dual-branch Vision Transformer together with cross-attention and graph-pooling mechanisms to integrate MRI and PET information, thereby enhancing complementary representations across imaging modalities. Ge et al. (2023) introduced MDMA, where cross-modal channel-spatial attention and cross-modal cross-attention are designed to enable interactive fusion between MRI and PET features. Zhang et al. (2023) developed MCAD, which separately encodes sMRI, FDG-PET, and CSF biomarkers and then integrates imaging and non-imaging information through a cross-modal attention module to strengthen inter-modality relationships. Lei et al. (2024) further modeled local and global interactions among different modalities in a unified feature space by combining feature-induced learning with a dual-level graph neural network. These studies demonstrate that cross-modal attention can effectively alleviate the coarse fusion problem caused by simple concatenation and improve the collaborative representation of multi-source information. Nevertheless, existing multimodal AD methods mainly focus on the fusion of MRI–PET, MRI–CSF, MRI–APOE, or cognitive features, while the exploitation of clinical text as a high-level disease-semantic modality remains insufficient. Clinical text contains rich semantic information related to medical history, chief complaints, cognitive status, and diagnostic reasoning; however, it does not naturally correspond to local structural changes in MRI in a one-to-one spatial manner. Therefore, how to stably inject clinical semantics into MRI representations without introducing erroneous fine-grained matching, and further achieve constrained fusion with anatomical priors, remains a critical challenge. To address these limitations, this paper proposes AFCG-Net, a unified framework that jointly models MRI structural representations, clinical textual semantics, and anatomical priors to improve discriminability, robustness, and medical interpretability in AD staging.

## 3 Methods

In this section, we present AFCG-Net, an anatomy-guided and frequency-aware cross-modal network for joint diagnosis using Alzheimer's disease (AD) MRI and clinical text. In AD, structural abnormalities are typically manifested as continuous neurodegenerative changes, including hippocampal and parahippocampal atrophy, ventricular enlargement, temporal cortical thinning, and gray matter loss. These alterations involve both subtle local cues and large-scale structural remodeling, making a single visual representation insufficient for reliable stage-aware classification.

Clinical text provides complementary high-level semantic information for disease staging, but direct image–text matching may weaken anatomically meaningful signals. To address this issue, we develop a three-stage collaborative framework in which structural imaging features, clinical semantics, and anatomical priors are jointly modeled. Specifically, ASFG enhances visual representations through frequency-aware multi-scale encoding, CASF performs cross-modal alignment under the guidance of global clinical semantics, and AGDF achieves gated deep fusion between anatomical priors and cross-modal features. Overall, AFCG-Net is designed to improve both AD stage recognition and the correspondence between brain structural abnormalities and clinically meaningful semantic information, thereby providing a more trustworthy and clinically valuable multimodal solution for MRI-assisted AD diagnosis.

### 3.1 Overall framework

**Figure 1:**
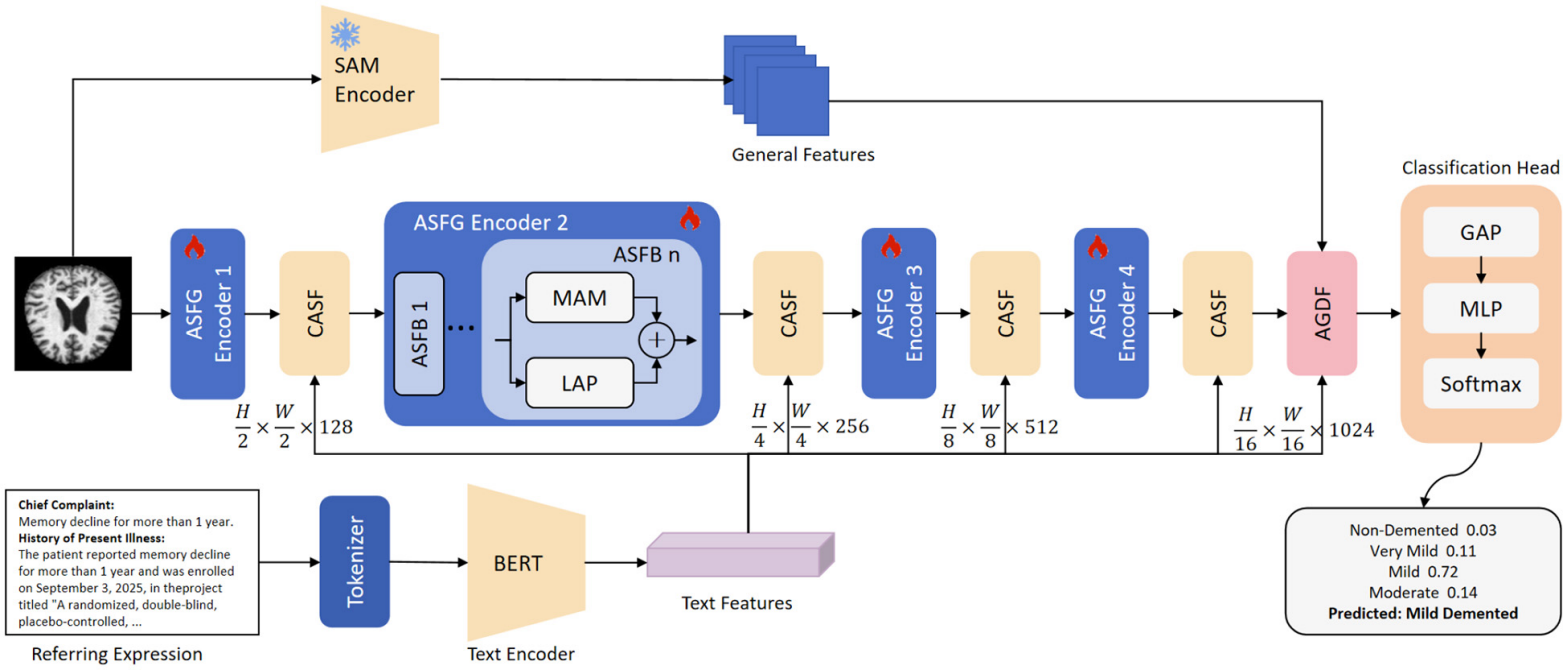
Overall architecture of AFCG-Net. Structural MRI and diagnostic text are jointly modeled through three stages: anatomy-guided visual encoding (ASFG), cross-modal semantic alignment (CASF), and anatomy-guided gated deep fusion (AGDF), followed by AD stage prediction.

The overall architecture of AFCG-Net is illustrated in Figure 1. The network takes MRI images *M* and diagnostic text *R* as joint inputs, and is composed of three consecutive stages: ASFG, CASF, and AGDF, which correspond to anatomy-guided visual representation learning, cross-modal semantic alignment, and diagnosis-constrained deep fusion, respectively. Rather than merely increasing architectural complexity, this design aims to jointly characterize AD-related structural abnormalities, clinically meaningful semantic information, and their intrinsic correspondence within a unified framework, thereby improving both diagnostic reliability and medical interpretability.

In the anatomy-aware encoding stage, the MRI image *M* is simultaneously fed into a trainable ASFG main branch and a frozen SAM-based structural prior branch. The ASFG branch extracts four-stage multi-scale visual features ${\left\{F_{i}^{A}\right\}}_{i=1}^{4}$, which are used to characterize imaging patterns closely related to AD staging, including weak local boundaries, regional atrophic changes, and global anatomical remodeling. In parallel, the SAM branch produces corresponding structural priors ${\left\{S_{i}\right\}}_{i=1}^{4}$, providing relatively stable boundary and morphological cues for subsequent feature interaction. Meanwhile, the diagnostic text *R* is mapped by a text encoder into a clinical semantic representation *T*, which supplements high-level disease information that cannot be explicitly captured by MRI alone.

In the cross-modal coarse semantic alignment stage, CASF takes ${\left\{F_{i}^{A}\right\}}_{i=1}^{4}$ and *T* as inputs and establishes a global constraint relationship between clinical text and MRI visual features through a frequency-aware semantic alignment mechanism. Combined with structured feature sampling, high-level semantic context is progressively injected into visual representations at different scales, yielding cross-modal aligned features ${\left\{F_{i}^{C}\right\}}_{i=1}^{4}$. The goal of this stage is not to enforce overly fine-grained object-level correspondence, but to use the overall clinical semantics provided by diagnostic text to guide multi-scale neurodegenerative patterns in a stable and consistent manner, thereby enhancing the robustness of vision–text collaborative modeling.

In the anatomy-guided gated deep fusion stage, AGDF integrates the cross-modal aligned features ${\left\{F_{i}^{C}\right\}}_{i=1}^{4}$ produced by CASF with the structural priors ${\left\{S_{i}\right\}}_{i=1}^{4}$ from the SAM branch under anatomical constraints, generating fused representations ${\left\{F_{i}^{G}\right\}}_{i=1}^{4}$. Through this process, the model is able to preserve key anatomical information while more effectively combining clinical semantics with imaging evidence, so that the final representation becomes not only more discriminative but also more consistent with medical reasoning. Finally, the classification head aggregates the fused multi-stage features and outputs the AD stage prediction ŷ.

Overall, AFCG-Net establishes a unified analytical pipeline from structural abnormality characterization to stage prediction through the collaborative modeling of anatomical priors, clinical semantic information, and multi-scale MRI representations. Beyond improving classification performance, the proposed framework emphasizes the correspondence between brain structural changes and clinically meaningful diagnostic semantics, thereby providing a more interpretable and potentially clinically valuable solution for MRI-assisted AD diagnosis.

The overall inference process of AFCG-Net can be summarized as follows, as in Equations 1–4:


$$\begin{array}{l} {\left\{F_{i}^{A}\right\}}_{i=1}^{4}=ASFG\left(M\right),\;{\left\{S_{i}\right\}}_{i=1}^{4}=SAM\left(M\right),\;T=Enc_{t}\left(R\right), \end{array}$$ (1)

$$\begin{array}{l} {\left\{F_{i}^{C}\right\}}_{i=1}^{4}=CASF\left({\left\{F_{i}^{A}\right\}}_{i=1}^{4},T\right), \end{array}$$ (2)

$$\begin{array}{l} {\left\{F_{i}^{G}\right\}}_{i=1}^{4}=AGDF\left({\left\{F_{i}^{C}\right\}}_{i=1}^{4},{\left\{S_{i}\right\}}_{i=1}^{4}\right), \end{array}$$ (3)

$$\begin{array}{l} ŷ=Cls\left({\left\{F_{i}^{G}\right\}}_{i=1}^{4}\right), \end{array}$$ (4)

where *Enc*_*t*_(·) denotes the text encoder, *Cls*(·) denotes the classification head, and ŷ represents the predicted diagnostic category of Alzheimer's disease. Functionally, ASFG, CASF, and AGDF are responsible for high-quality visual representation learning, stable cross-modal semantic alignment, and anatomy-constrained deep collaborative fusion, respectively. Together, they form the core technical pathway of AFCG-Net for MRI-assisted AD stage prediction.

### 3.2 ASFG: anatomy-guided spatial-frequency representation learning

The key discriminative cues in Alzheimer's disease MRI do not usually appear as explicit lesions with clear boundaries or isolated shapes. Instead, they are more often reflected in a continuum of neurodegenerative changes, including hippocampal and parahippocampal atrophy, ventricular enlargement, temporal cortical thinning, and gray matter volume loss. These abnormalities are distributed not only in weak local boundaries and subtle regional morphological variations, but also in large-scale global brain structural remodeling, thus requiring cross-scale representation learning. Meanwhile, MRI is often affected by low contrast, intensity inhomogeneity, and complex textural interference, making it difficult for conventional single-scale convolutions or purely local region modeling strategies to simultaneously preserve fine-grained structural sensitivity and global degeneration awareness.

Motivated by these observations, we propose the anatomy-guided spatial-frequency representation module, denoted as ASFG, which jointly models multi-scale anatomical patterns in the spatial domain and low-frequency-guided information in the frequency domain during the main visual encoding stage. The purpose of this design is to obtain visual representations that are better aligned with AD stage discrimination. In essence, spatial-domain modeling emphasizes local boundary preservation, regional morphological characterization, and hierarchical structural relationships, whereas frequency-domain modeling helps suppress texture noise and spurious local variations that may interfere with clinically relevant representation learning. Through this joint mechanism, ASFG is able to retain sensitivity to structural details while more stably capturing the overall progression trend of brain degeneration.

Specifically, after ASFG encoding, the network outputs multi-scale visual features ${\left\{F_{i}^{A}\right\}}_{i=1}^{4}$ at the *i*-th stage, which serve as stable and anatomically discriminative inputs for the subsequent cross-modal coarse semantic alignment. Different from conventional visual encoders that rely primarily on local texture responses, ASFG places greater emphasis on the continuous expression and hierarchical modeling of AD-related structural abnormalities. This provides a more reliable foundation for the subsequent integration of MRI-derived structural evidence with clinically meaningful textual semantics.

Specifically, at the *i*-th stage, given the input feature:


$$F_{i}^{\text{in}}\in {\mathbb{R}}^{C_{i}\times H_{i}\times W_{i}},$$

ASFG is composed of *N*_*i*_ cascaded anatomy-guided spatial-frequency blocks, and outputs the final stage representation through a group-wise residual scheme. The initial input is defined as:


$$F_{i,0}=F_{i}^{\text{in}}.$$

For the *n*-th block, the incoming feature is first normalized to improve the stability of subsequent multi-branch modeling:


$${\bar{F}}_{i,n}=\text{Norm}\left(F_{i,n-1}\right).$$

**Figure 2:**
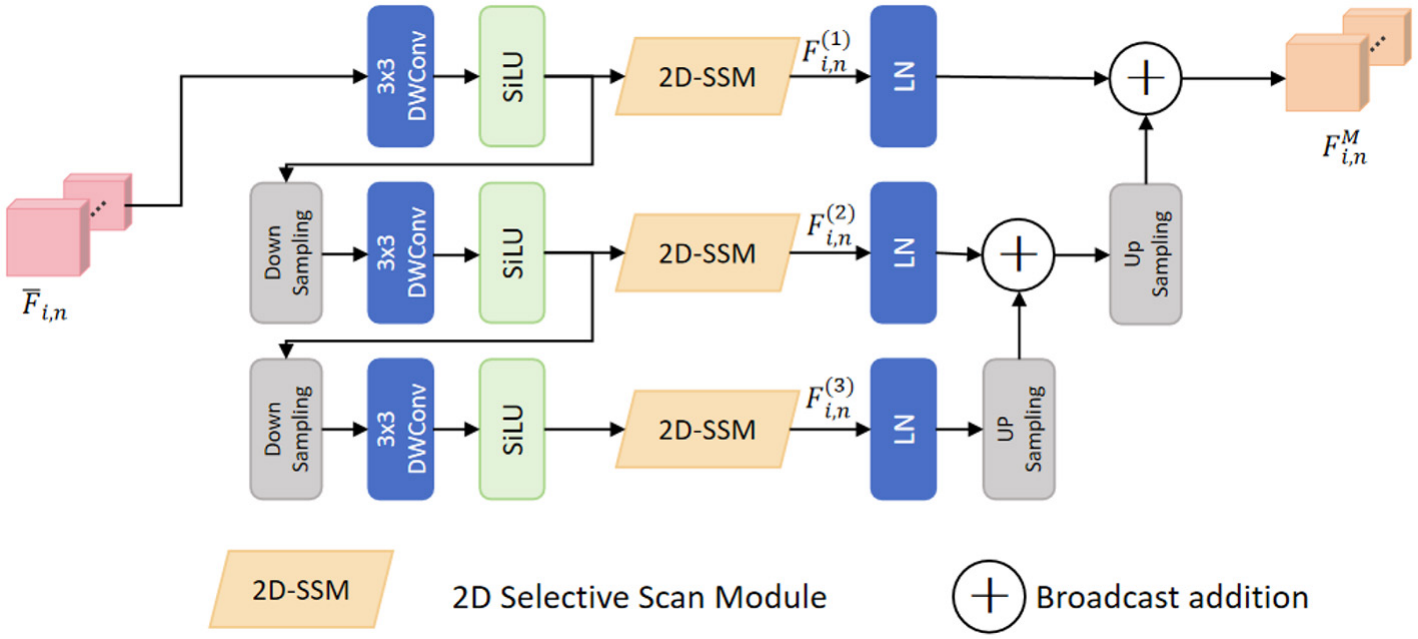
Architecture of the multi-scale atrophy modeling (MAM) module. MAM captures AD-related structural patterns at multiple spatial scales through parallel branches with different downsampling rates, and adaptively fuses them into a unified representation.

**Figure 3:**
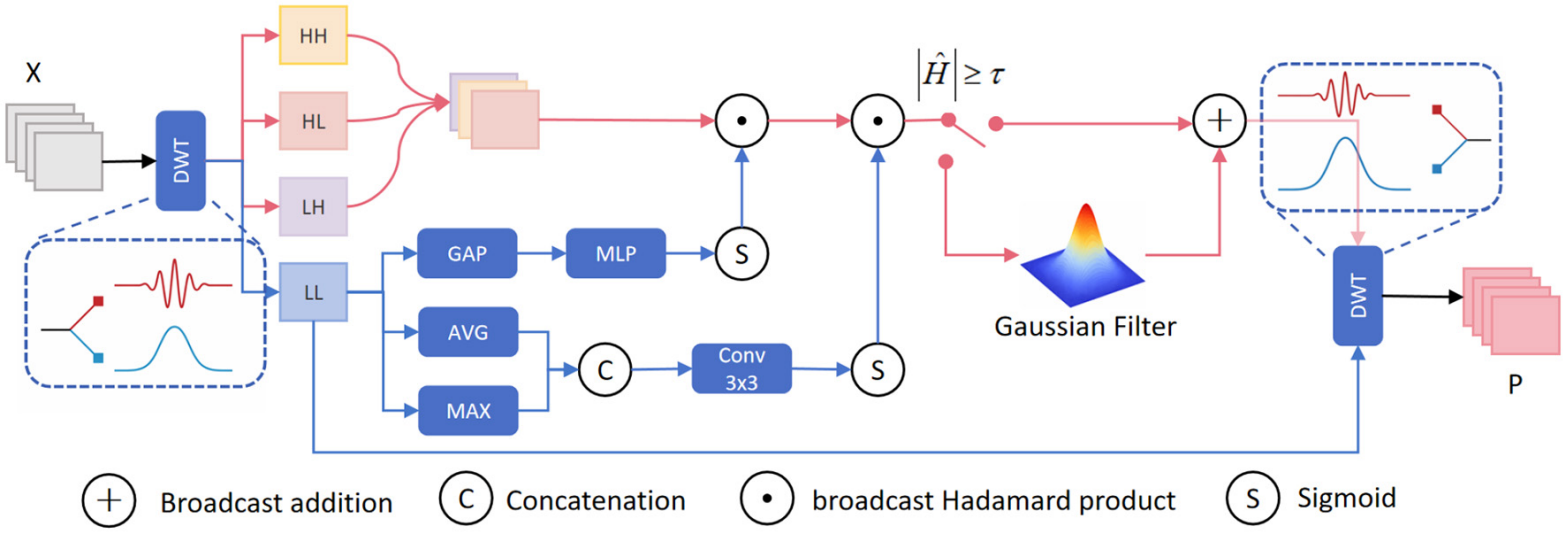
Architecture of the low-frequency anatomy purification (LAP) module. LAP decomposes the input feature into one low-frequency component and three directional high-frequency sub-bands, uses the low-frequency component to guide gating, and reconstructs a more stable anatomical representation.

The normalized feature ${\bar{F}}_{i,n}$ is then fed in parallel into the multi-scale atrophy modeling branch (MAM) and the low-frequency anatomy purification branch (LAP). The former is designed to capture AD-related structural abnormalities as continuous neurodegenerative patterns across different spatial scales, whereas the latter uses low-frequency guidance to suppress texture noise and spurious local perturbations, thereby improving the stability and medical interpretability of structural representation learning. The detailed architectures of MAM and LAP are shown in Figures 2, 3, respectively.

In parallel with MAM, the LAP branch is designed to further purify visual representations in the frequency domain, so as to reduce the influence of low-contrast imaging, textural interference, and spurious local variations on structural discrimination. In Alzheimer's disease MRI, diagnostically meaningful cues are not simply reflected by enhanced high-frequency textures, but rather by the stable expression of anatomical boundaries, morphological contours, and structural continuity. Therefore, if representation learning relies solely on spatial-domain feature extraction, irrelevant fine-grained textures may be incorporated together with true neurodegenerative signals, thereby weakening the model's focus on key anatomical information. To address this issue, LAP explicitly separates low-frequency anatomical components from multi-directional high-frequency details through wavelet decomposition, and uses low-frequency information to guide and constrain the high-frequency responses, thus achieving frequency-domain purification of structural representation learning.

Specifically, given the input feature ${\bar{F}}_{i,n}$, LAP first decomposes it into one low-frequency anatomical component and three directional high-frequency sub-bands by wavelet transform, as in Equation 5:


$$\begin{array}{l} \left[L_{i,n},H_{i,n}^{h},H_{i,n}^{v},H_{i,n}^{d}\right]=\text{W}\left({\bar{F}}_{i,n}\right), \end{array}$$ (5)

where *L*_*i, n*_ denotes the low-frequency anatomical component, and $H_{i,n}^{h}$, $H_{i,n}^{v}$, and $H_{i,n}^{d}$ denote the horizontal, vertical, and diagonal high-frequency sub-bands, respectively. Since the low-frequency component preserves more stable structural contours and regional tissue information, we use *L*_*i, n*_ to generate both spatial and channel-wise gates for regulating the retention of subsequent high-frequency responses as in Equation 6:


$$\begin{array}{l} A_{i,n}^{s}=\sigma \left(\text{Con}{\text{v}}_{s}\left(L_{i,n}\right)\right),\;A_{i,n}^{c}=\sigma \left(\text{MLP}\left(\text{GAP}\left(L_{i,n}\right)\right)\right). \end{array}$$ (6)

On top of this, to prevent unstable texture noise in the high-frequency sub-bands from interfering with structural modeling, we further introduce a bandwidth-controlled Gaussian smoothing operator to generate frequency-confidence gates for the three directional sub-bands as in formula Equation 7:


$$\begin{array}{l} G_{i,n}^{k}=\sigma \left(\Gamma_{b}\left(H_{i,n}^{k}\right)\right),\;k\in \left\{h,v,d\right\}, \end{array}$$ (7)

and apply joint constraints to the directional high-frequency responses as in Equation 8:


$$\begin{array}{l} {Ĥ}_{i,n}^{k}=A_{i,n}^{s}⊙A_{i,n}^{c}⊙G_{i,n}^{k}⊙H_{i,n}^{k}. \end{array}$$ (8)

In this way, LAP preserves structural boundaries and morphological details while suppressing random texture perturbations that are unrelated to disease degeneration patterns, making the high-frequency information more consistent with anatomically meaningful expression.

The low-frequency component and the refined high-frequency sub-bands are then fed into the inverse wavelet transform to produce the output of the LAP branch as in Equation 9:


$$\begin{array}{l} F_{i,n}^{L}={\text{W}}^{-1}\left(L_{i,n},{Ĥ}_{i,n}^{h},{Ĥ}_{i,n}^{v},{Ĥ}_{i,n}^{d}\right). \end{array}$$ (9)

After obtaining the outputs of the MAM and LAP branches, a lightweight gating network is used to adaptively estimate their relative importance as in Equation 10:


$$\begin{array}{l} \left[\alpha_{i,n},\beta_{i,n}\right]=\text{Softmax}\left(\text{MLP}\left(\text{GAP}\left(\text{Concat}\left(F_{i,n}^{M},F_{i,n}^{L}\right)\right)\right)\right), \end{array}$$ (10)

and the current block output is updated in a residual manner as in Equation 11:


$$\begin{array}{l} F_{i,n}=F_{i,n-1}+\alpha_{i,n}F_{i,n}^{M}+\beta_{i,n}F_{i,n}^{L}. \end{array}$$ (11)

Accordingly, the final output of ASFG at the *i*-th stage is denoted as Equation 12:


$$\begin{array}{l} F_{i}^{A}=F_{i,N_{i}}. \end{array}$$ (12)

In summary, ASFG unifies multi-scale neurodegenerative pattern modeling, low-frequency-guided frequency purification, and residual feature updating within the main visual encoding stage. As a result, the resulting features ${\left\{F_{i}^{A}\right\}}_{i=1}^{4}$ become jointly sensitive to local anatomical boundaries, regional morphological variations, and global structural degeneration. This provides a more stable and anatomically discriminative visual basis for the subsequent consistency modeling between MRI-derived structural evidence and clinically meaningful textual semantics in CASF.

### 3.3 CASF: cross-modal coarse semantic alignment and structural fusion

Although ASFG provides anatomically discriminative visual features, such features alone do not fully resolve the semantic gap between structural MRI manifestations and clinically expressed diagnostic knowledge.

After obtaining the visual features ${\left\{F_{i}^{A}\right\}}_{i=1}^{4}$, it remains necessary to further address the consistency between MRI-derived structural representations and clinical text semantics. Different from referring expressions in natural image understanding, diagnostic text for Alzheimer's disease usually emphasizes high-level clinical semantics, such as disease progression, cognitive scale assessments, and physicians' diagnostic judgments, rather than providing explicit target categories or spatial location cues. Meanwhile, abnormalities in MRI are characterized by cross-scale distribution, weak local boundaries, and continuous neurodegenerative evolution. Therefore, directly enforcing fine-grained object-level matching may introduce alignment bias and even weaken anatomically meaningful discriminative information.

**Figure 4:**
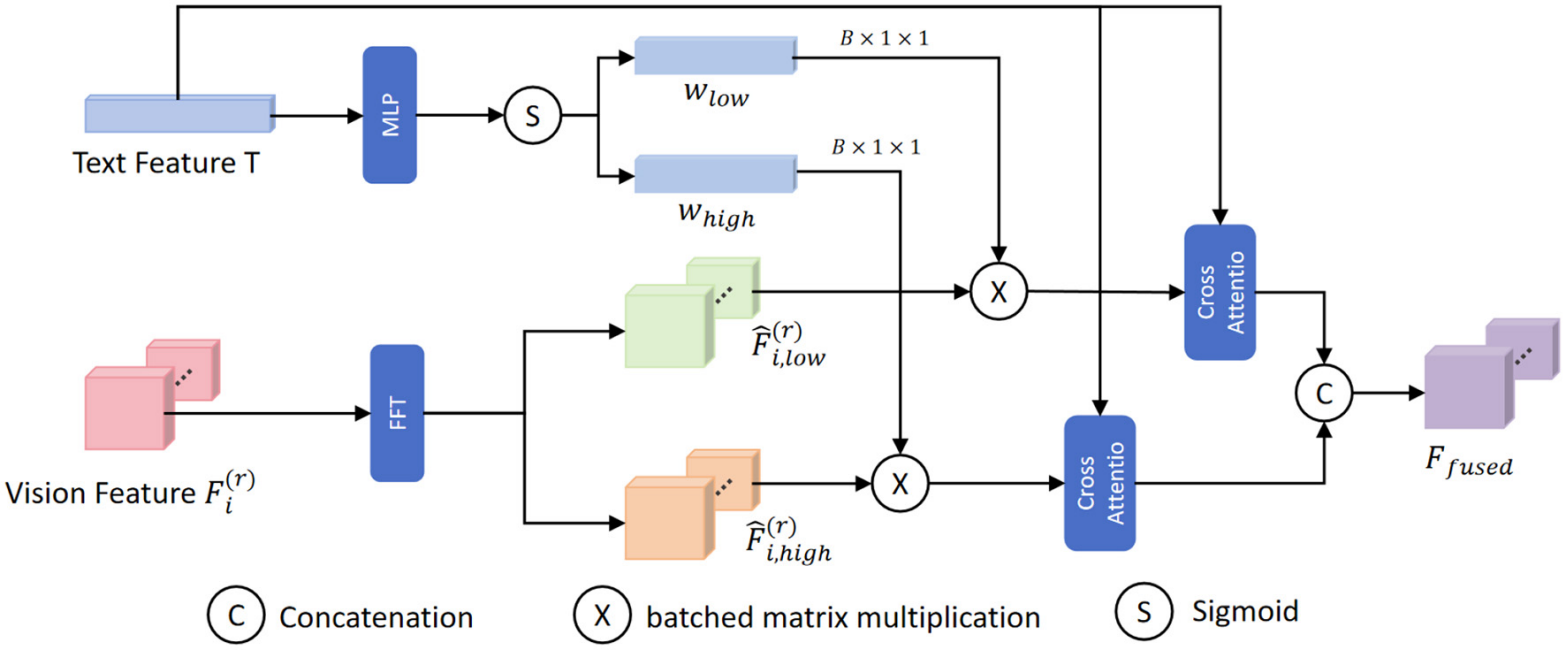
Architecture of the cross-modal alignment and semantic fusion (CASF) module. CASF aligns MRI features with clinical text semantics through frequency-aware decomposition and cross-modal fusion.

Motivated by this observation, we propose the cross-modal alignment and semantic fusion module (CASF), as shown in Figure 4 which performs frequency-aware cross-modal alignment on multi-scale visual features $F_{i}^{A}$ under the guidance of global clinical semantics *T*. Rather than pursuing overly precise position-level correspondence, CASF emphasizes the use of overall disease semantics from diagnostic text to provide stable guidance for neurodegenerative patterns at different spatial scales, thereby improving the consistency between visual features and clinical semantics and forming a more reliable cross-modal representation for subsequent anatomy-constrained fusion.

Specifically, given the visual feature produced by ASFG at the *i*-th stage,


$$F_{i}^{A}\in {\mathbb{R}}^{B\times C_{i}\times H_{i}\times W_{i}},$$

and the global text feature *T*, CASF first constructs *R* scale mappings from $F_{i}^{A}$, denoted as Equation 13:


$$\begin{array}{l} F_{i}^{A,\left(r\right)}=\psi_{r}\left(F_{i}^{A}\right),\;r=1,2,\ldots ,R, \end{array}$$ (13)

where ψ_*r*_(·) denotes the feature mapping function at the *r*-th scale. Then, each scale feature is transformed by fast Fourier transform and decomposed into low-frequency and high-frequency components as in Equation 14:


$$\begin{array}{l} \left[F_{i,\text{low}}^{A,\left(r\right)},F_{i,\text{high}}^{A,\left(r\right)}\right]=\text{Split}\left(\text{FFT}\left(F_{i}^{A,\left(r\right)}\right)\right). \end{array}$$ (14)

In the text branch, the global clinical semantic feature *T* is first projected by a multilayer perceptron, followed by a Sigmoid activation to generate frequency gating vectors, which are then split into low-frequency and high-frequency gates as in Equation 15:


$$\begin{array}{l} \left[w_{\text{low}},w_{\text{high}}\right]=\text{Split}\left(\sigma \left(\text{MLP}\left(T\right)\right)\right),\;w_{\text{low}},w_{\text{high}}\in {\mathbb{R}}^{B\times 1\times 1}. \end{array}$$ (15)

These two gating weights are then used to modulate the low-frequency and high-frequency visual features, respectively as in Equation 16:


$$\begin{array}{l} {\hat{F}}_{i,\text{low}}^{\left(r\right)}=w_{\text{low}}⊙F_{i,\text{low}}^{A,\left(r\right)},\;{\hat{F}}_{i,\text{high}}^{\left(r\right)}=w_{\text{high}}⊙F_{i,\text{high}}^{A,\left(r\right)}. \end{array}$$ (16)

On top of this, the two frequency branches are separately fed into cross-attention modules, where the text feature *T* serves as semantic guidance for coarse-grained alignment as in Equation 17:


$$\begin{array}{l} {\tilde{F}}_{i,\text{low}}^{\left(r\right)}=\text{CA}\left({\hat{F}}_{i,\text{low}}^{\left(r\right)},T\right),\;{\tilde{F}}_{i,\text{high}}^{\left(r\right)}=\text{CA}\left({\hat{F}}_{i,\text{high}}^{\left(r\right)},T\right), \end{array}$$ (17)

where CA(·) denotes the cross-attention operation. This dual-branch design allows the low-frequency pathway to preserve global anatomical consistency, while the high-frequency pathway maintains fine-grained boundary variations and local structural differences, enabling textual semantics to be injected into visual representations in a more stable and anatomically coherent manner.

The aligned low-frequency and high-frequency branches are then concatenated along the channel dimension to form the fused feature at the current scale as in Equation 18:


$$\begin{array}{l} F_{i,\text{fused}}^{\left(r\right)}=\text{Concat}\left({\tilde{F}}_{i,\text{low}}^{\left(r\right)},{\tilde{F}}_{i,\text{high}}^{\left(r\right)}\right). \end{array}$$ (18)

Finally, the fused features from all scales are restored to a unified spatial resolution and aggregated to produce the stage output of CASF as in Equation 19:


$$\begin{array}{l} F_{i}^{C}=\text{Mixer}\left(\text{Conca}{\text{t}}_{r=1}^{R}\left(\text{Up}\left(F_{i,\text{fused}}^{\left(r\right)}\right)\right)\right), \end{array}$$ (19)

where Up(·) denotes the upsampling operation, and Mixer(·) denotes channel integration and feature remapping.

In summary, CASF injects the global clinical semantics *T* into the visual features ${\left\{F_{i}^{A}\right\}}_{i=1}^{4}$ through a unified process of frequency decomposition, text-guided gating, dual-branch cross-attention, and multi-scale aggregation, yielding the cross-modally aligned features ${\left\{F_{i}^{C}\right\}}_{i=1}^{4}$. This process not only preserves the continuous anatomical response patterns in MRI, but also substantially strengthens the consistency between visual representation and disease semantics, thereby establishing a more robust cross-modal basis for the subsequent anatomy-guided deep fusion in AGDF.

### 3.4 AGDF: anatomy-guided gated deep fusion

**Figure 5:**
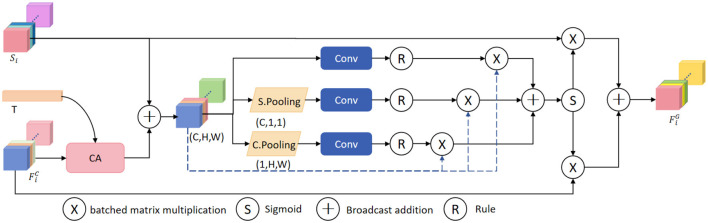
Architecture of the anatomy-guided gated deep fusion (AGDF) module. AGDF fuses cross-modal features, anatomical priors, and clinical semantics through gated deep fusion.

Although CASF has produced cross-modally aligned features ${\left\{F_{i}^{C}\right\}}_{i=1}^{4}$ under the guidance of global disease semantics, these features remain primarily driven by semantic relevance and still provide insufficient explicit constraints for stable anatomical patterns, such as hippocampal boundaries, ventricular contours, and cortical thickness variations. In contrast, the frozen SAM branch provides structural priors ${\left\{S_{i}\right\}}_{i=1}^{4}$ with stronger boundary consistency and morphological stability, but lacks direct guidance from task-relevant disease semantics. Therefore, an effective collaboration between anatomical priors and cross-modal semantics becomes critical for improving the final diagnostic decision. To address this issue, we propose the anatomy-guided gated deep fusion module (AGDF), which performs gated collaborative fusion of $F_{i}^{C}$, *S*_*i*_, and the global text semantics *T* under anatomical constraints. As shown in Figure 5, AGDF coordinates structural information, semantic information, and local detail responses simultaneously in both channel and spatial dimensions through text-aware interaction, three-branch attention modeling, and dynamic reweighting, yielding the fused representations ${\left\{F_{i}^{G}\right\}}_{i=1}^{4}$.

Specifically, given the CASF output feature $F_{i}^{C}$, the SAM structural prior *S*_*i*_, and the global text semantic feature *T* at the *i*-th stage, the text feature is first projected to the current visual scale through a linear mapping, yielding the stage-specific textual representation as in Equation 20:


$$\begin{array}{l} F_{i}^{T}=\phi_{i}\left(T\right), \end{array}$$ (20)

where ϕ_*i*_(·) denotes the scale-adaptive text projection function. Then, a cross-attention module is used to construct text-enhanced semantic features as in Equation 21:


$$\begin{array}{l} F_{i}^{CT}=CA\left(F_{i}^{C},F_{i}^{T}\right), \end{array}$$ (21)

where *CA*(·) denotes the cross-attention operation.

Subsequently, the text-enhanced feature $F_{i}^{CT}$ is combined with the structural prior *S*_*i*_ through broadcast addition to obtain an intermediate fused representation as in Equation 22:


$$\begin{array}{l} F_{i}^{M}=S_{i}⊕F_{i}^{CT}, \end{array}$$ (22)

where ⊕ denotes element-wise broadcast addition. This fused feature is then fed into three parallel branches to model local detail response, spatial response, and channel response, respectively as in Equations 23–25:


$$\begin{array}{l} F_{i}^{LD}=\delta \left(\text{Conv}\left(F_{i}^{M}\right)\right)⊗F_{i}^{M}, \end{array}$$ (23)

$$\begin{array}{l} F_{i}^{SA}=\delta \left(\text{Conv}\left(g_{s}\left(F_{i}^{M}\right)\right)\right)⊗F_{i}^{M}, \end{array}$$ (24)

$$\begin{array}{l} F_{i}^{CH}=\delta \left(\text{Conv}\left(g_{c}\left(F_{i}^{M}\right)\right)\right)⊗F_{i}^{M}, \end{array}$$ (25)

where *g*_*s*_(·) and *g*_*c*_(·) denote spatial average pooling and channel average pooling, respectively, δ(·) denotes non-linear rule-based mapping, and ⊗ denotes element-wise multiplication.

The responses of the three branches are then summed and activated by Sigmoid to generate an anatomy-semantic joint gating map as in Equation 26:


$$\begin{array}{l} W_{i}=\sigma \left(F_{i}^{LD}+F_{i}^{SA}+F_{i}^{CH}\right). \end{array}$$ (26)

Finally, this gating map is used to complementarily reweight the two feature streams, producing the output of AGDF as in Equation 27:


$$\begin{array}{l} F_{i}^{G}=W_{i}⊗S_{i}+\left(1-W_{i}\right)⊗F_{i}^{C}. \end{array}$$ (27)

In this formulation, *W*_*i*_ emphasizes the stable anatomy-aware responses provided by SAM, whereas (1−*W*_*i*_) preserves the semantically discriminative multimodal cues from CASF. In this way, AGDF achieves dynamic collaborative fusion of anatomical boundaries, global disease semantics, and local degenerative details without explicitly amplifying irrelevant textures or scanning noise.

Overall, AGDF performs gated deep collaborative fusion between the cross-modally aligned features ${\left\{F_{i}^{C}\right\}}_{i=1}^{4}$ and the structural priors ${\left\{S_{i}\right\}}_{i=1}^{4}$ under anatomical constraints, resulting in fused representations ${\left\{F_{i}^{G}\right\}}_{i=1}^{4}$. This process unifies structural information, disease semantics, and local detail responses through adaptive reweighting, enabling the final classification decision to be built upon more stable and coherent multi-source representations, and thereby providing direct support for downstream AD stage prediction ŷ.

### 3.5 Class-balanced focal loss

In Alzheimer's disease (AD) staging, the numbers of samples across different diagnostic categories are often highly imbalanced. Meanwhile, the neuroanatomical differences between adjacent disease stages are relatively subtle, especially between non-dementia and very mild dementia, where feature overlap frequently occurs. Under such circumstances, directly optimizing the standard cross-entropy loss may cause the training process to be dominated by majority classes and easily classified samples, thereby weakening the model's ability to learn from minority classes, transitional-stage samples, and low-confidence hard examples. To alleviate this issue, we adopt the class-balanced focal loss as the final classification objective. Specifically, the class-balanced term compensates for the sample-frequency discrepancy among different diagnostic categories in the training set, while the focal modulation term reduces the loss contribution of easily classified samples, encouraging the model to focus more on hard examples and boundary-ambiguous early AD stages.

Given the fused features ${\left\{F_{i}^{G}\right\}}_{i=1}^{4}$ produced by AGDF, the classification head outputs the logits $z_{b}\in {\mathbb{R}}^{K}$ for the *b*-th sample, where *K* = 4 denotes the four diagnostic categories. The predicted probability corresponding to the ground-truth class *y*_*b*_ is defined as Equation 28:


$$\begin{array}{l} p_{b,y_{b}}=\frac{\exp\left(z_{b,y_{b}}\right)}{\sum_{j=1}^{K}\exp\left(z_{b,j}\right)}. \end{array}$$ (28)

Let *n*_*c*_ denote the number of samples belonging to class *c* in the original training set. The class-balanced weight is computed according to the effective number of samples as in Equation 29:


$$\begin{array}{l} \alpha_{c}=\frac{1-\beta }{1-\beta^{n_{c}}},\;{\tilde{\alpha }}_{c}=\frac{K\alpha_{c}}{\sum_{j=1}^{K}\alpha_{j}}, \end{array}$$ (29)

where β∈(0, 1) controls the strength of class-frequency compensation. For a mini-batch of size *B*, the final class-balanced focal loss is formulated as Equation 30:


$$\begin{array}{l} L_{\text{CBF}}=-\frac{1}{B}\overset{B}{\underset{b=1}{\sum }}{\tilde{\alpha }}_{y_{b}}{\left(1-p_{b,y_{b}}\right)}^{\gamma }\log\left(p_{b,y_{b}}+\epsilon \right), \end{array}$$ (30)

where γ is the focal modulation factor and ϵ is a small constant introduced to ensure numerical stability. The class weights are computed only from the original training set before data augmentation and are not applied to the validation or testing sets, ensuring that model evaluation faithfully reflects the true data distribution.

## 4 Experiments

### 4.1 Dataset and preprocessing

This study employs an MRI-text dataset constructed from a self-constructed dataset and the public Alzheimer's Disease Neuroimaging Initiative (ADNI) cohort. The self-constructed dataset included 10 de-identified clinical text samples provided by Dazhou Central Hospital and additional data collected from publicly available platforms such as Kaggle, comprising 2,106 paired samples of structural MRI scans and clinical text. According to the clinical diagnostic records, these samples were categorized into four diagnostic groups: Non-dementia, Very Mild dementia, Mild dementia, and Moderate dementia. To improve the external generalizability of the proposed model, ADNI was further incorporated as a multi-center public validation source. For ADNI, only T1-weighted structural MRI and baseline demographic/clinical variables were used, without introducing additional modalities such as PET, CSF, or APOE genotype. ADNI1, ADNI GO, ADNI2, and the in-house cohort were jointly used for model development, whereas ADNI3 was reserved as an independent external validation cohort. The diagnostic labels in ADNI were mapped to the four-class setting of this study according to a unified rule: CN, MCI, and AD were mapped to Non-dementia, Very Mild dementia, and Mild dementia, respectively. Since no independent Moderate dementia category was available in the adopted ADNI statistics, ADNI samples were not assigned to the Moderate dementia category. Samples with missing key diagnostic or staging information were excluded.

Clinical text was constructed using a structured template to ensure input consistency across different data sources. For each sample, the textual description consisted of sex, age, years of education, marital status, and chief complaint, following the format: “Sex: [sex]. Age: [age] years. Education: [education] years. Marital status: [marital status]. Chief complaint: [chief complaint].” For the in-house cohort, textual information was extracted from de-identified electronic medical records. The chief complaints were summarized by clinical staff into symptom-level phrases, while identity-related information, administrative content, and expressions directly revealing the final diagnostic category were removed. For the ADNI cohort, demographic information was obtained from baseline records. Since ADNI does not provide routine outpatient-style free-text chief complaints, chief complaints were not manually generated from diagnostic labels. When explicit memory concerns reported by participants or informants were available in the baseline assessment, they were standardized into controlled phrases; otherwise, the field was uniformly filled as “Chief complaint: not available in ADNI.” Missing demographic fields were denoted as “unknown.” All textual fields were restricted to information available at or before the corresponding MRI acquisition.

All MRI scans were processed using a unified preprocessing pipeline, including spatial normalization, bias-field correction, brain extraction, intensity normalization to [0, 1], and resizing to 224 × 224 pixels. Contrast-limited adaptive histogram equalization (CLAHE) was then applied to enhance local contrast, followed by ESRGAN to improve the visibility of weak boundaries and fine-grained anatomical structures. Data splitting was performed at the subject level to prevent sample leakage from the same subject. The in-house cohort and the ADNI1/GO/2 development cohorts were stratified by diagnostic category and data source, and were split into training, validation, and internal test sets with a ratio of 8:1:1. ADNI3 was used exclusively for external validation. Class imbalance was addressed only during training through class-balanced batch sampling, class-balanced focal loss, and mild data augmentation for minority-class MRI samples. The validation set, internal test set, and ADNI3 external validation set retained their original class distributions without resampling or augmentation, ensuring that the evaluation results faithfully reflected the real data distribution. The distribution of the original non-augmented samples across cohorts is reported in Table 1.

**Table 1:** Distribution of samples across the self-collected and ADNI cohorts.

Cohort	Non-dementia	Very Mild dementia	Mild dementia	Moderate dementia	Total	Usage
Self-collected cohort	842	631	422	211	2,106	Internal evaluation
ADNI1/GO/2 development cohort	892	837	118	0	1,847	Internal evaluation
ADNI3 external validation cohort	98	122	24	0	244	External validation
Total	1,832	1,590	564	211	4,197	–

Values denote original, non-augmented samples after inclusion, exclusion, label harmonization, and MRI–text pairing. For ADNI, CN, MCI, and AD were mapped to Non-dementia, Very Mild dementia, and Mild dementia, respectively. Since no independent Moderate dementia category was available in the adopted ADNI statistics, the Moderate dementia count in ADNI was set to 0. Augmented samples were used only during training and were not counted in this table.

### 4.2 Evaluation metrics

For MRI-based Alzheimer's disease stage recognition, a reasonable and comprehensive evaluation protocol is essential for objectively assessing model performance. Since this task concerns not only overall classification accuracy but also the ability to distinguish different disease stages, especially the subtle differences between early and adjacent stages, we adopted Accuracy, Precision, Recall, F1-score, and the confusion matrix as evaluation metrics.

Accuracy measures the overall consistency between model predictions and ground-truth labels, and is defined as Equation 31:


$$\begin{array}{l} \text{Accuracy}=\frac{TP+TN}{TP+TN+FP+FN}. \end{array}$$ (31)

Precision measures the proportion of correctly predicted positive samples among all predicted positive samples. In computer-aided medical diagnosis, this metric reflects the false-positive level; a higher Precision indicates that positive predictions made by the model are more trustworthy. It is defined as Equation 32:


$$\begin{array}{l} \text{Precision}=\frac{TP}{TP+FP}. \end{array}$$ (32)

Recall measures the ability of the model to identify truly positive samples, that is, the proportion of actual positive samples that are correctly detected. In early AD screening and risk identification, Recall is particularly important because missed detections may directly reduce the clinical utility of the model. It is defined as Equation 33:


$$\begin{array}{l} \text{Recall}=\frac{TP}{TP+FN}. \end{array}$$ (33)

The F1-score is the harmonic mean of Precision and Recall, and reflects the balance between prediction reliability and positive detection capability. It is defined as Equation 34:


$$\begin{array}{l} \text{F}1=2\times \frac{\text{Precision}\times \text{Recall}}{\text{Precision}+\text{Recall}}. \end{array}$$ (34)

In addition, we incorporated the confusion matrix to further characterize the model's classification behavior across different stages of Alzheimer's disease, thereby elucidating potential patterns of misclassification among the Non-dementia, Very Mild dementia, Mild dementia, and Moderate dementia categories. Compared with a single scalar metric, the confusion matrix provides a more intuitive assessment of the model's ability to discriminate between adjacent disease stages, and thus offers complementary evidence for subsequent result interpretation and discussion of clinical relevance. Meanwhile, to further improve the statistical rigor of the study, 95% confidence intervals were reported for all evaluation metrics. These intervals not only indicate the plausible range within which the true metric values may lie, but also provide additional statistical support for the reliability and robustness of the proposed model.

### 4.3 Training and testing analysis

**Figure 6:**
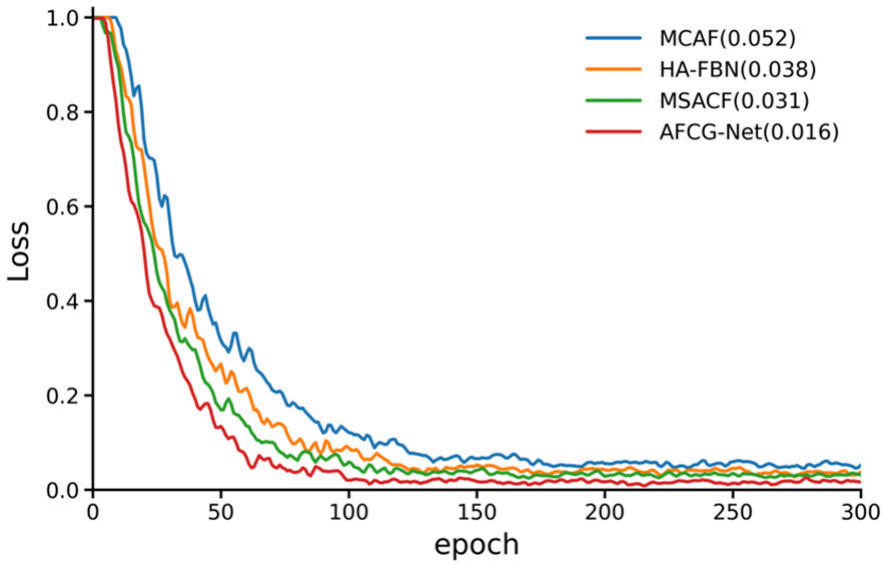
Loss curves of different methods during training.

To evaluate the optimization capability, convergence stability, and predictive reliability of AFCG-Net, we compare it with three representative multimodal methods, namely MCAF, HA-FBN, and MSACF. As shown in Figure 6, all models exhibit a clear decreasing trend in training loss during the early optimization stage, whereas their convergence behaviors differ considerably. AFCG-Net achieves a faster loss reduction and eventually converges to the lowest loss value of 0.016. In contrast, the final loss values of MSACF, HA-FBN, and MCAF are 0.031, 0.038, and 0.052, respectively. These results indicate that AFCG-Net can more effectively reduce the residual classification error during parameter optimization, leading to a more stable and reliable training process.

**Figure 7:**
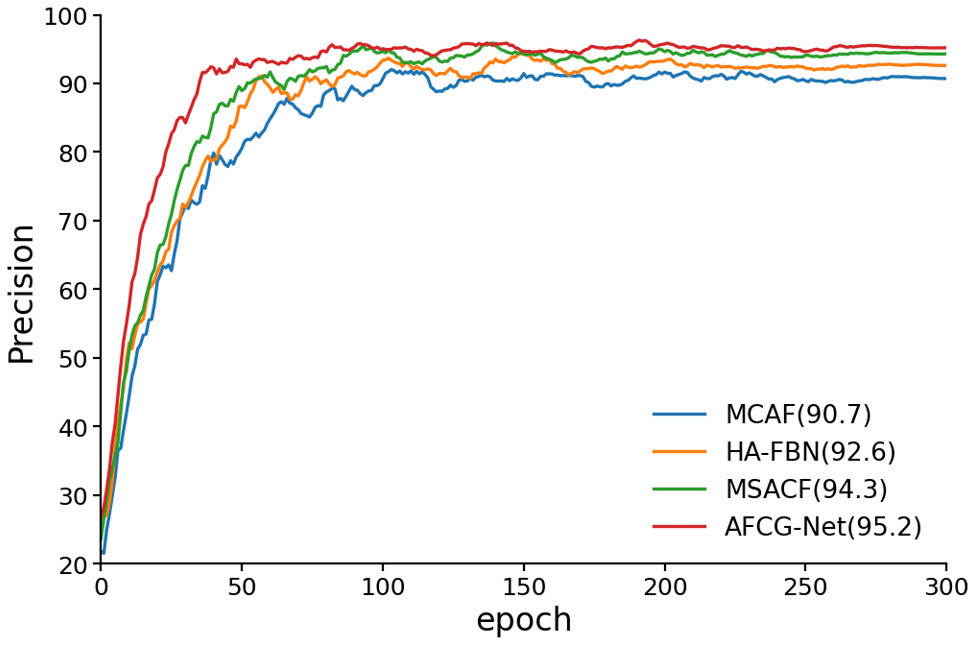
Accuracy curves of different methods during training.

The precision curves further confirm the predictive advantage of AFCG-Net. As shown in Figure 7, AFCG-Net improves rapidly during the early training stage and maintains a consistently high and stable precision in the later stage, finally reaching 95.2%. This performance surpasses MSACF, HA-FBN, and MCAF, which achieve final precision values of 94.3%, 92.6%, and 90.7%, respectively. These results suggest that, by jointly modeling anatomical structural priors, clinical semantic constraints, and gated deep fusion, AFCG-Net learns more stable and discriminative representations, thereby improving the reliability of AD staging prediction.

**Figure 8:**
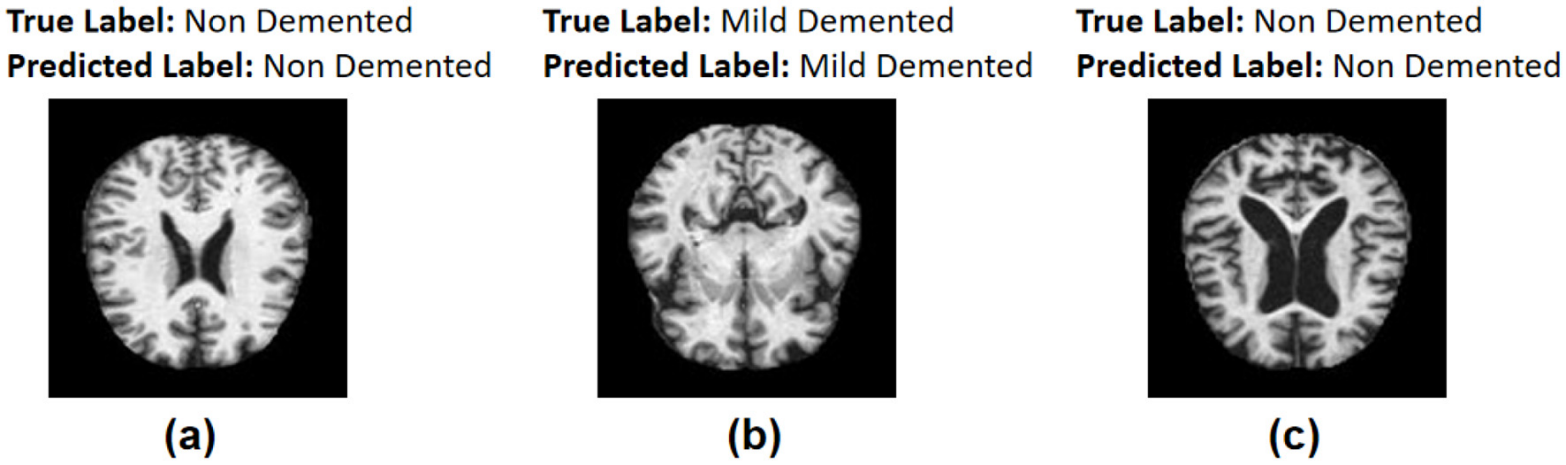
**(a–c)** Representative correctly classified cases.

**Figure 9:**
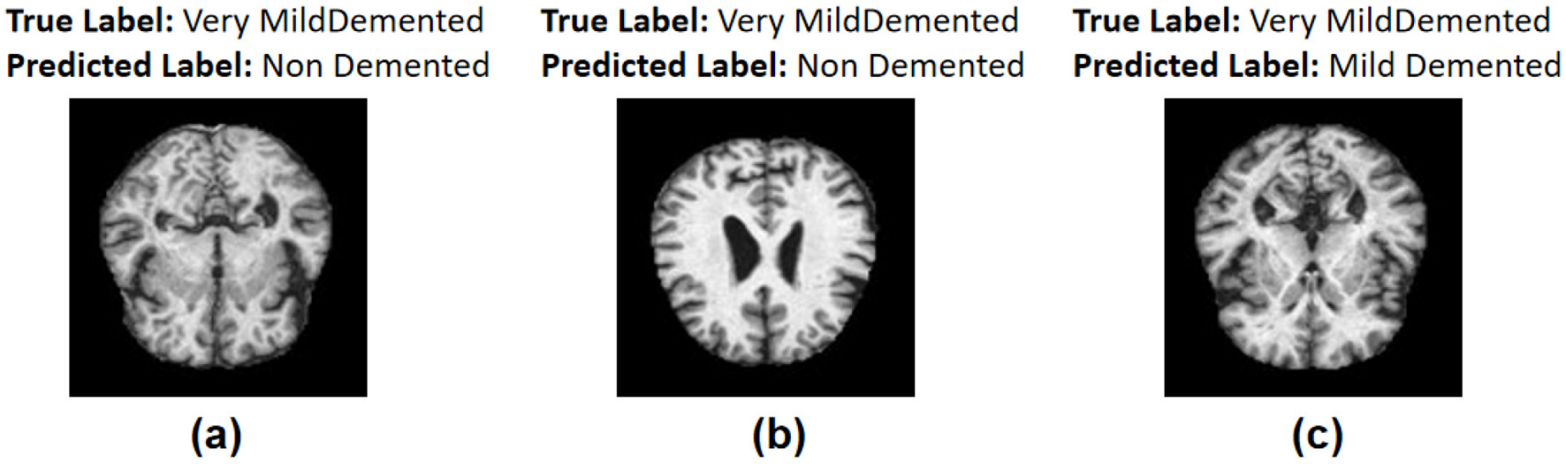
**(a–c)** Representative misclassified cases. The true and predicted labels are shown in each panel.

**Figure 10:**
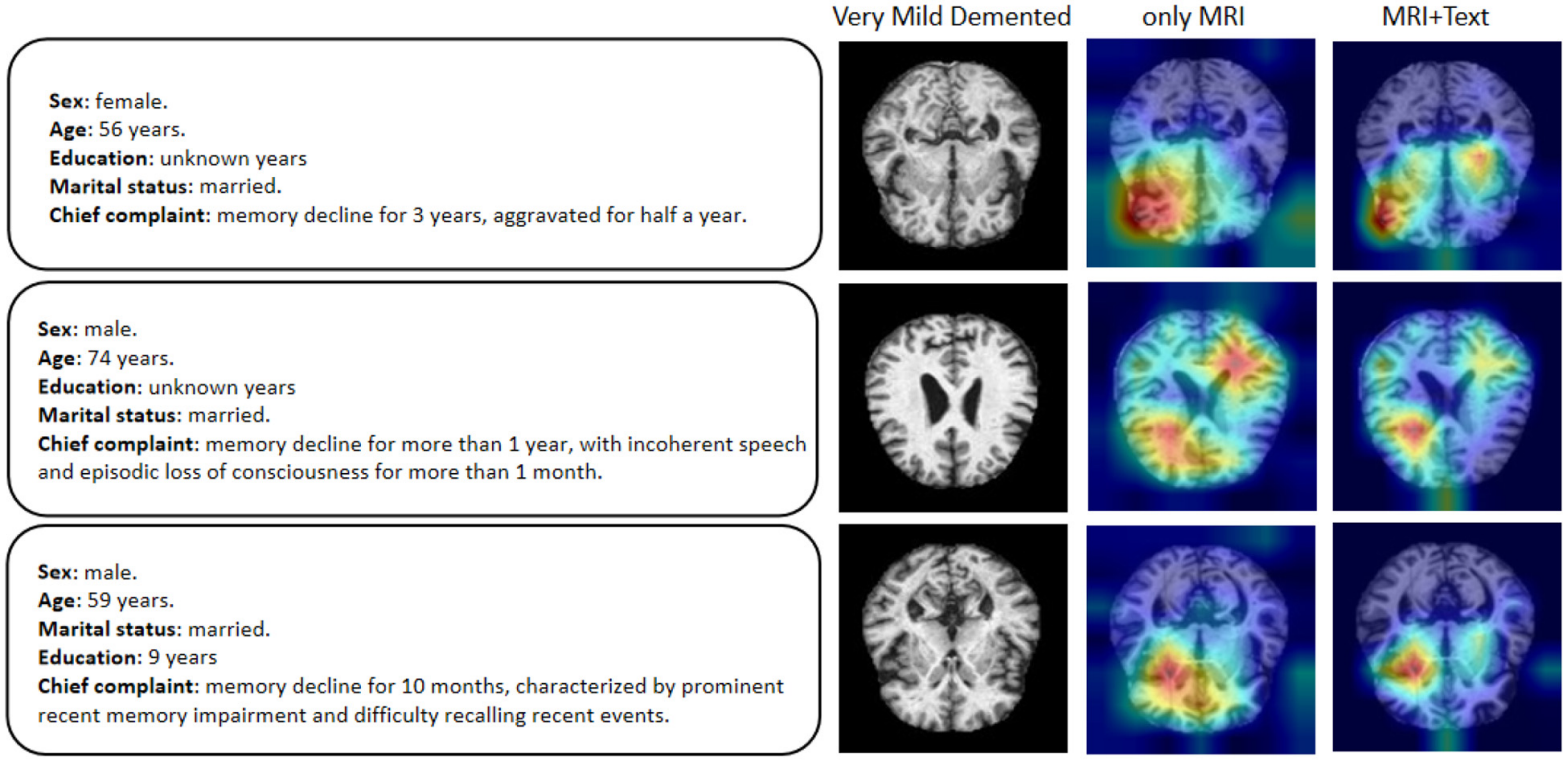
Comparison of heatmap responses for misclassified samples under the MRI-only model and the MRI+Text model.

To further analyze the classification behavior of the proposed model, representative correctly classified samples are shown in Figure 8, representative misclassified samples are presented in Figure 9, and the corresponding heatmap responses are illustrated in Figure 10. As can be observed, the model is able to distinguish Non-dementia and Mild dementia cases relatively reliably, indicating that it has learned structurally discriminative patterns with reasonable stability. In contrast, most misclassifications are concentrated in the Very Mild dementia category, with samples often being incorrectly assigned to either the Non-dementia or Mild dementia groups. This finding suggests that the Very Mild dementia stage still exhibits substantial representational overlap with its adjacent categories, making it the most challenging class in the current task.

Further comparison of the heatmaps indicates that the MRI-only model tends to exhibit relatively diffuse response patterns, whereas the introduction of textual semantics leads to more concentrated attention, particularly over anatomically relevant regions associated with disease progression. This finding suggests that clinical text provides effective global semantic guidance for image-based discrimination, thereby improving the model's sensitivity to subtle neurodegenerative alterations in early-stage AD. Overall, these visualization results are well aligned with the quantitative findings and further validate the effectiveness of joint MRI–text modeling.

### 4.4 Ablation study

To validate the contribution of each constituent module, we conducted a progressive ablation study under a unified training setting, and the results are reported in Table 2. The baseline model adopts a Mamba backbone, achieving Precision, Recall, and F1-score values of 92.6%, 93.2%, and 92.4%, respectively.

**Table 2:** Ablation study of the constituent components of AFCG-Net.

Method	SAM	BERT	ASFG	CASF	AGDF	Precision (%)	Recall (%)	F1-score (%)
mamba (baseline)						92.6	93.2	92.4
SAM	✓					92.8	93.1	92.6
BERT		✓				94.7	94.5	94.2
SAM+BERT	✓	✓				95.1	94.8	94.5
SAM+BERT+ASFG	✓	✓	✓			95.6	95.3	95.1
SAM+BERT+CASF	✓	✓		✓		95.3	94.9	94.7
SAM+BERT+AGDF	✓	✓			✓	95.2	94.8	94.9
SAM+BERT+ASFG+CASF	✓	✓	✓	✓		96.1	95.8	95.4
SAM+BERT+ASFG+AGDF	✓	✓	✓		✓	95.8	95.7	95.7
SAM+BERT+CASF+AGDF	✓	✓		✓	✓	95.4	95.2	95.3
SAM+BERT+ASFG+CASF+AGDF	✓	✓	✓	✓	✓	**96.3**	**96.5**	**95.8**

Bold values indicate the best performance among all compared methods.

When SAM is introduced alone into the baseline model, the F1-score increases only slightly from 92.4% to 92.6%, suggesting that the independent gain provided by structural priors is limited. By contrast, introducing BERT alone raises the F1-score to 94.2%, indicating that clinical textual semantics contribute more directly to disease discrimination. When SAM and BERT are incorporated simultaneously, the F1-score is further improved to 94.5%, which suggests that anatomical priors and textual semantics provide complementary information.

On top of the SAM+BERT setting, the inclusion of the proposed core modules further improves model performance. Among them, ASFG yields the most pronounced gain, increasing the F1-score to 95.1%, which indicates that anatomy-guided spatial-frequency modeling is a key factor for performance improvement. CASF and AGDF improve the F1-score to 94.7% and 94.9%, respectively, demonstrating that both cross-modal semantic constraint and anatomy-guided gated fusion can effectively enhance feature representation. Furthermore, among the dual-module combinations, ASFG+AGDF achieves the best performance, with an F1-score of 95.7%, outperforming ASFG+CASF (95.4%) and CASF+AGDF (95.3%). This finding suggests that under stronger visual representation support, the effect of deep fusion can be more fully realized.

When ASFG, CASF, and AGDF are all incorporated, the complete model achieves the best overall results, with Precision, Recall, and F1-score reaching 96.3%, 96.5%, and 95.8%, respectively, corresponding to improvements of 3.7, 3.3, and 3.4 percentage points over the baseline. These results indicate that the three modules form an effective collaborative chain across visual representation learning, semantic constraint, and deep fusion, jointly supporting the performance gains of AFCG-Net.

### 4.5 Comparison with state-of-the-art methods

To further evaluate the classification performance of AFCG-Net, we compare it with several representative methods recently developed for AD diagnosis, as reported in Table 3. The compared methods cover conventional imaging-based classification models, machine learning approaches, and recent multimodal frameworks, including MCAF (Nan et al., 2022), ECSNet (Xin et al., 2023), MCAN (Zhang et al., 2023), MDMA (Ge et al., 2023), AMFS (Zhang et al., 2024a), HA-FBN (Zhang et al., 2024a), and MSACF Liu et al. (2026). Overall, AFCG-Net achieves the best or tied-best performance across all four diagnostic categories. For the Non-dementia category, AFCG-Net obtains a precision of 95.8%, a recall of 95.4%, and an F1-score of 95.6%. For the Very Mild dementia category, it achieves 95.2%, 94.8%, and 95.0% in precision, recall, and F1-score, respectively. For both Mild dementia and Moderate dementia, AFCG-Net reaches 100% across all evaluation metrics. Compared with the strong baseline MSACF, AFCG-Net improves the F1-score by 1.1 and 0.9 percentage points for Non-dementia and Very Mild dementia, respectively, demonstrating its more stable discriminative capability in early-stage and boundary-ambiguous categories.

**Table 3:** Comparison with representative methods for AD classification.

References	Method	Class	Precision (%)	Recall (%)	F1-score (%)
Saim and Amel (2022)	InceptionV3 feature, and the random forest classifier (2022)	Non-demented	77.7	77.5	77.6
		Mild demented	92.5	91.8	92.1
		Moderate demented	96.6	96.2	96.4
		Very mild demented	76.4	75.9	76.1
Hajamohideen et al. (2023)	CNN with triplet-loss (2023)	Non-demented	92.8	92.5	92.6
		Mild demented	98.6	98.1	98.3
		Moderate demented	100	100	100
		Very mild demented	91.5	91.2	91.3
Nan et al. (2022)	Multimodal multi-classification assessment framework (MCAF) (2022)	Non-demented	92.8	92.4	92.6
		Mild demented	99.2	98.8	99.0
		Moderate demented	100	100	100
		Very mild demented	90.7	90.4	90.5
Xin et al. (2023)	Efficient Conv-Swin Net for sMRI-based AD diagnosis (ECSNet) (2023)	Non-demented	94.3	93.6	93.9
		Mild demented	99.8	98.4	99.1
		Moderate demented	100	100	100
		Very mild demented	91.8	91.3	91.5
Zhang et al. (2023)	Multi-modal cross-attention network for sMRI, FDG-PET, and CSF data (MCAN) (2023)	Non-demented	92.3	91.9	92.1
		Mild demented	99.1	98.8	98.9
		Moderate demented	100	100	100
		Very mild demented	91.2	90.8	91.0
Lal et al. (2024)	Optimized Machine Learning Model (EEG-based Analysis) (2024)	Non-demented	92.1	91.8	91.9
		Mild demented	98.6	98.7	98.6
		Moderate demented	100	100	100
		Very mild demented	91.8	91.5	91.6
Ge et al. (2023)	Multi-attention multimodal AD diagnosis model using MRI, PET, and clinical data (2023)	Non-demented	93.2	92.8	93.0
		Mild demented	99.1	98.6	98.8
		Moderate demented	100	100	100
		Very mild demented	91.8	91.4	91.6
Zhang et al. (2024b)	Feature-aware multimodal auto-fusion framework (AMFS) (2024)	Non-demented	94.1	93.7	93.9
		Mild demented	97.9	97.6	97.7
		Moderate demented	100	100	100
		Very mild demented	92.5	92.1	92.3
Chen et al. (2024)	Graph Neural Network (GNN) Node Classification (2024)	Non-demented	90.8	90.3	90.5
		Mild demented	96.7	96.2	96.4
		Moderate demented	100	100	100
		Very mild demented	88.7	88.4	88.5
Zhang et al. (2024a)	Hierarchical attentive functional brain network model (HA-FBN) (2024)	Non-demented	93.8	93.1	93.4
		Mild demented	98.9	98.2	98.5
		Moderate demented	100	100	100
		Very mild demented	92.6	91.9	92.2
Wang et al. (2024)	Hybrid Multimodal Deep Learning Model (2024)	Non-demented	86.2	85.6	85.9
		Mild demented	95.6	95.2	95.4
		Moderate demented	100	100	100
		Very mild demented	84.9	84.6	84.7
Nasra and Gupta (2025)	DenseNet (2025)	Non-demented	92.1	91.6	91.8
		Mild demented	98.3	98.1	98.2
		Moderate demented	100	100	100
		Very mild demented	91.2	90.7	90.9
Liu et al. (2026)	Multimodal sMRI–APOE classification framework (MSACF) (2026)	Non-demented	94.7	94.4	94.5
		Mild demented	95.4	94.8	95.1
		Moderate demented	100	100	100
		Very mild demented	94.3	93.9	94.1
This work	AFCG-Net	Non-demented	95.8	95.4	95.6
		Mild demented	100	100	100
		Moderate demented	100	100	100
		Very mild demented	95.2	94.8	95.0

In the two most challenging categories, Non-dementia and Very Mild dementia, AFCG-Net achieved F1-scores of 95.5% and 93.5%, respectively, both surpassing the best existing results by a clear margin. This finding indicates that the proposed model is more effective at capturing fine-grained differences between normal aging and early pathological changes. Collectively, these results show that AFCG-Net not only preserves its advantage in middle and later disease stages, but also delivers improved recognition performance in difficult categories, highlighting its overall robustness and potential clinical utility.

### 4.6 Visualization

**Figure 11:**
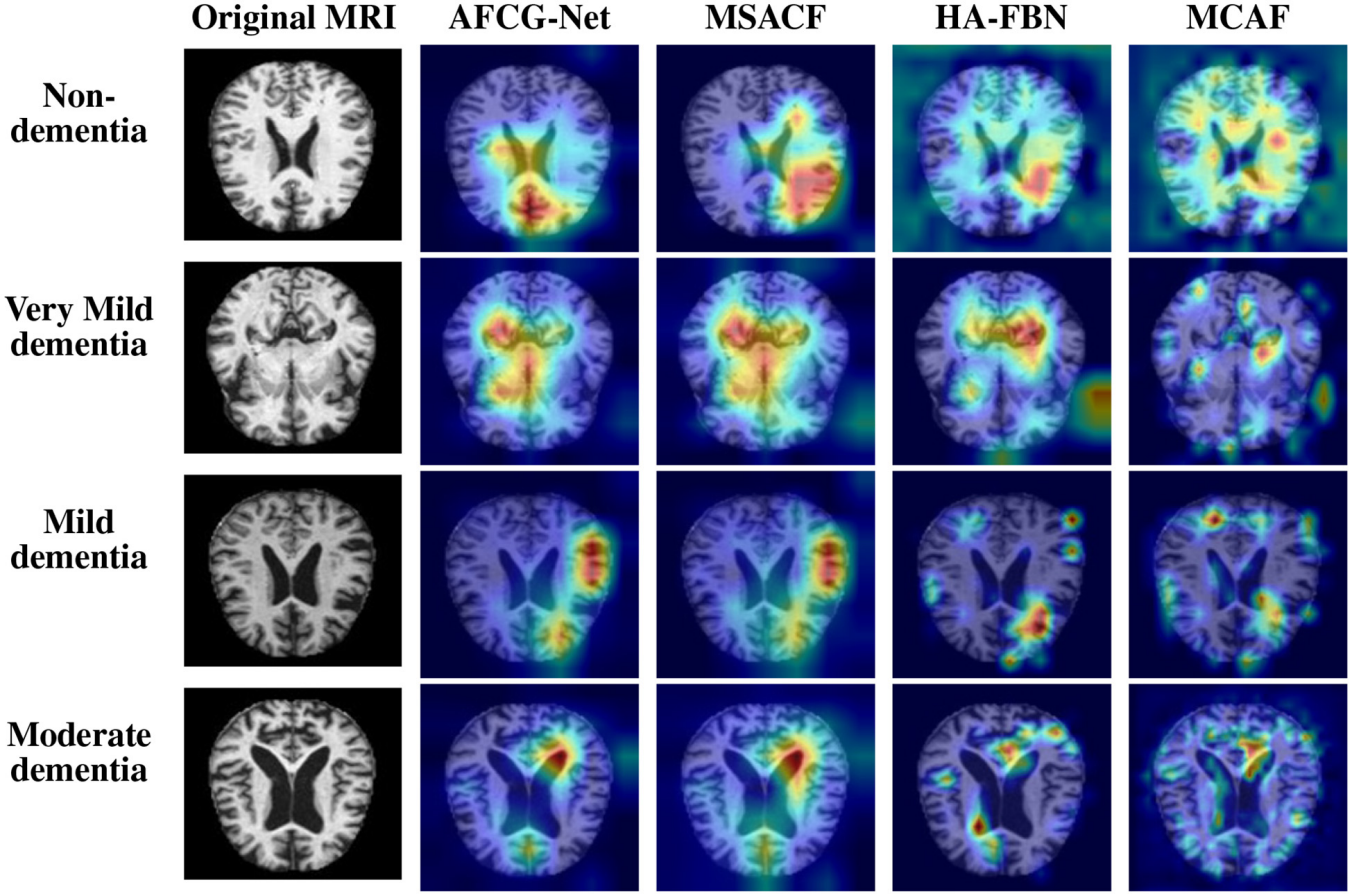
Grad-CAM comparison of different models on four-category Alzheimer's disease samples. The first column shows the original MRI images, and the remaining columns present the corresponding activation maps generated by AFCG-Net, MSACF, HA-FBN, and MCAF, respectively. The rows correspond to Non-dementia, Very Mild dementia, Mild dementia, and Moderate dementia samples.

To analyze the discriminative basis of different models, Grad-CAM was employed to visualize the response regions of DenseNet169, AD-ResNet50, LSTM, and AFCG-Net, as shown in Figure 11. Overall, the high-response regions of DenseNet169 and AD-ResNet50 are relatively diffuse and exhibit evident non-discriminative activation patterns. The responses of LSTM are comparatively more concentrated, but remain mainly focused around the central axis. In contrast, AFCG-Net produces more compact activation regions, which are primarily distributed around the ventricles and the bilateral medial temporal regions, thereby showing clearer structural relevance.

A closer inspection across different disease stages further shows that AFCG-Net can capture subtle early structural alterations in Non-dementia and Very Mild dementia cases, while more consistently focusing on regions associated with ventricular enlargement and temporal lobe atrophy in Mild dementia and Moderate dementia cases. This indicates that, by jointly modeling anatomical priors, clinical semantics, and multi-scale MRI representations, AFCG-Net can suppress irrelevant texture responses and guide model decisions toward imaging patterns that are more consistent with AD-related neurodegeneration. These visualization results are consistent with the quantitative experimental findings, further validating the advantages of AFCG-Net in both classification performance and structural interpretability.

## 5 Conclusion

In this study, we proposed AFCG-Net, an anatomy-guided and frequency-aware cross-modal network for joint MRI–text diagnosis of Alzheimer's disease. By integrating anatomy-guided visual encoding, cross-modal semantic alignment, and structurally constrained deep fusion, AFCG-Net enables unified modeling of MRI-derived structural information, clinical text semantics, and anatomical priors. Experimental results showed that the proposed framework outperformed comparative methods in training stability, convergence behavior, and classification performance, with particularly stronger fine-grained discrimination in the challenging Non-dementia and Very Mild dementia categories. Grad-CAM visualizations further indicated that AFCG-Net more consistently focused on anatomically relevant regions associated with disease progression, supporting both its discriminative ability and structural interpretability. Nevertheless, Very Mild dementia remains the most challenging stage. Future work will focus on multi-center external validation and the integration of 3D MRI, longitudinal follow-up data, and richer clinical information to further improve generalizability, early-stage sensitivity, and clinical applicability.

## Data Availability

The data analyzed in this study is subject to the following licenses/restrictions: “The datasets are not publicly available because they contain de-identified clinical MRI images and corresponding case-text data derived from hospital records. Access is restricted due to patient privacy concerns and institutional ethical requirements. De-identified data may be made available by the corresponding author upon reasonable request and with permission from the relevant institution.” Requests to access these datasets should be directed to FC, Department of Neurology, Dazhou Central Hospital, 962898357@qq.com.
